# Supplementary material for: Epidermal Barrier Function and Skin Homeostasis in Atopic Dermatitis: The Impact of Age
Source: Life (Basel). 2022 Jan 17;12(1):132. doi: 10.3390/life12010132 (PMC8779900; doi:10.3390/life12010132)
Supplement: Supplementary file 1 [file life-12-00132-s001.zip › life-1528964-supplementary.pdf]

Supplementary

Table S1. Homeostasis parameters between patients with atopic dermatitis and healthy volunteers.

Skin Homeostasis Parameters	Healthy Skin (n = 81)	Uninvolved AD Skin (n = 81)	AD Eczematous Lesion (n = 81)	*p*-Value *	*p*-Value **	*p*-Value ***
TEWL ($g \cdot m^{-2} \cdot h^{-1}$)	9.98 (6.59)	25.51 (20.55)	28.38 (18.13)	<0.001 *	<0.001 **	0.275
SCH (AU)	44.36 (19.57)	39.36 (15.67)	24.23 (17.14)	0.077	<0.001 **	<0.001 ***
Temperature (°C)	31.11 (1.64)	31.28 (1.33)	32.07 (1.31)	0.496	<0.001 **	<0.001 ***
Erythema (AU)	218.93 (54.33)	239.88 (68.23)	391.11 (81.17)	0.086	<0.001 **	<0.001 ***
pH	5.92 (0.63)	6.01 (0.72)	6.15 (0.69)	0.114	0.039 **	0.298
Elasticity (%)	0.77 (0.1)	0.74 (0.15)	0.69 (0.17)	0.219	0.014 **	0.038 ***

AD, atopic dermatitis; AU, arbitrary units; SCH, stratum corneum hydration; TEWL, transepidermal water loss. * *p*-value after using Student's *t*-test for independent samples to compare skin barrier function between healthy skin and uninvolved AD skin. ** *p*-value after using Student's *t*-test for independent samples to compare skin barrier function between healthy skin and AD eczematous lesions. *** *p*-value after using Student's *t*-test for paired samples to compare skin barrier function between uninvolved AD skin and AD eczematous lesions.

Table S2. Homeostasis parameters between patients with atopic dermatitis <30 and healthy participants <30 years of age.

Skin Homeostasis Parameters	Healthy Volunteers (n = 41)	Uninvolved AD Skin (n = 52)	AD Eczematous Lesion (n = 52)	*p*-Value *	*p*-Value **	*p*-Value ***
TEWL ($g \cdot m^{-2} \cdot h^{-1}$)	9.99 (7.38)	25.69 (20.51)	28.32 (15.68)	<0.001 *	<0.001 **	0.411
SCH (AU)	48.13 (24.90)	38.22 (15.97)	22.47 (14.70)	0.030 *	<0.001 **	<0.001 ***
Temperature (°C)	31.07 (1.87)	31.05 (1.49)	31.94 (1.28)	0.937	0.010 **	<0.001 ***
Erythema (AU)	226.73 (76.92)	226.75 (48.22)	388.80 (78.32)	0.999	<0.001 **	<0.001 ***
pH	5.81 (0.74)	6.16 (0.77)	6.23 (0.76)	0.029 *	0.008 **	0.228
Elasticity (%)	0.79 (0.10)	0.78 (0.11)	0.71 (0.16)	0.872	0.040 **	0.003 ***

AD, atopic dermatitis; AU, arbitrary units; SCH, stratum corneum hydration; TEWL, transepidermal water loss. * *p*-value after using Student's *t*-test for independent samples to compare skin barrier function between healthy participants and uninvolved AD skin in participants <30 years of age. ** *p*-value after using Student's *t*-test for independent samples to compare skin barrier function between healthy skin and AD eczematous lesions in participants <30 years of age. *** *p*-value after using Student's *t*-test for paired samples to compare skin barrier function between uninvolved AD skin and AD eczematous lesions in participants <30 years of age.

Table S3. Homeostasis parameters between patients with atopic dermatitis ≥30 and healthy participants ≥30 years of age.

Skin Homeostasis Parameters	Healthy Volunteers (n = 40)	Uninvolved AD Skin (n = 29)	AD Eczematous Lesion (n = 29)	*p*-Value *	*p*-Value **	*p*-Value ***
TEWL ($g{\cdot}m^{-2}{\cdot}h^{-1}$)	10.75 (5.57)	25.18 (20.98)	28.48 (22.17)	0.001 *	<0.001 **	0.483
SCH (AU)	43.16 (10.87)	41.56 (15.11)	27.63 (20.95)	0.638	<0.001 **	0.001 ***
Temperature (°C)	31.17 (1.24)	31.70 (0.86)	32.30 (1.36)	0.042 *	0.001 **	0.011 ***
Erythema (AU)	214.08 (56.85)	263.82 (40.51)	395.32 (88.46)	0.004 *	<0.001 **	<0.001 ***
pH	6.02 (0.51)	5.96 (0.59)	5.96 (0.48)	0.646	0.603	0.982
Elasticity (%)	0.74 (0.11)	0.65 (0.16)	0.65 (0.20)	0.043 *	0.040 **	0.782

AD, atopic dermatitis; AU, arbitrary units; SCH, stratum corneum hydration; TEWL, transepidermal water loss. * *p*-value after using Student's *t*-test for independent samples to compare skin barrier function between healthy skin and uninvolved AD skin in participants ≥30 years of age. ** *p*-value after using Student's *t*-test for independent samples to compare skin barrier function between healthy skin and AD eczematous lesions in participants ≥30 years of age. *** *p*-value after using Student's *t*-test for paired samples to compare skin barrier function between uninvolved AD skin and AD eczematous lesions in participants ≥30 years of age.

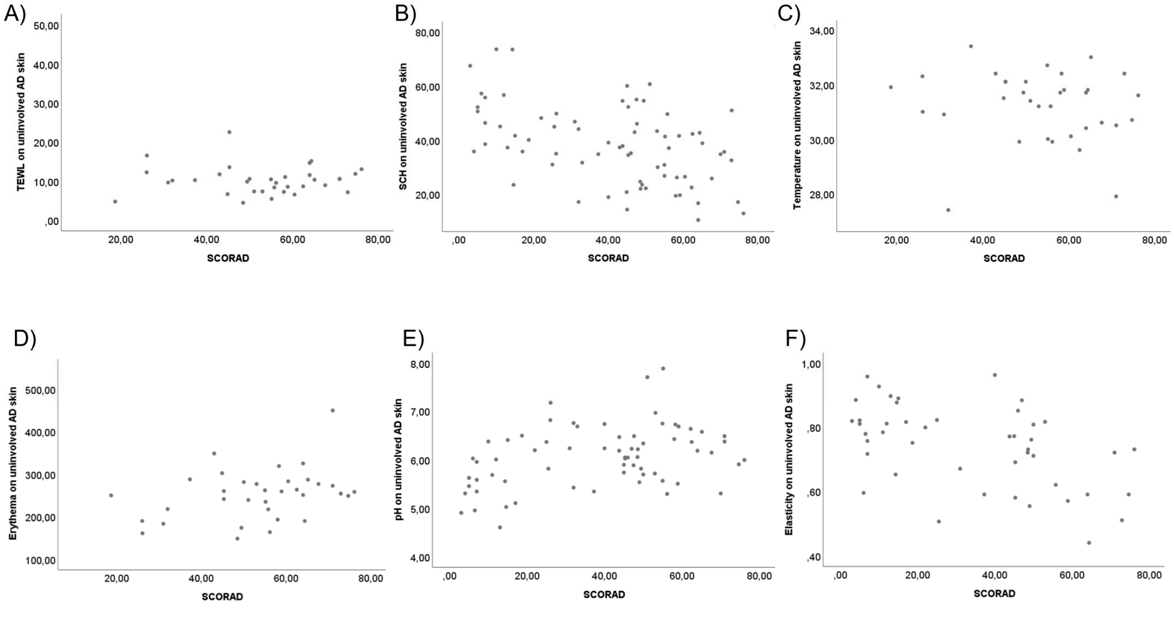

Figure S1. Correlation between disease severity and homeostasis parameters on uninvolved AD skin. (**A**) SCORAD and TEWL (r = −0.672, $p < 0.001$). (**B**) SCORAD and SCH (r = −0.504, $p < 0.001$). (**C**) SCORAD and temperature (r = 0.002, $p = 0.968$), (**D**) SCORAD and erythema (r = 0.250, $p = 0.090$), (**E**) SCORAD and pH (r = 0.401, $p = 0.001$), (**F**) SCORAD and elasticity (r = −0.541, $p < 0.001$). AD, atopic dermatitis; SCH, stratum corneum hydration; SCORAD, SCORing Atopic Dermatitis.

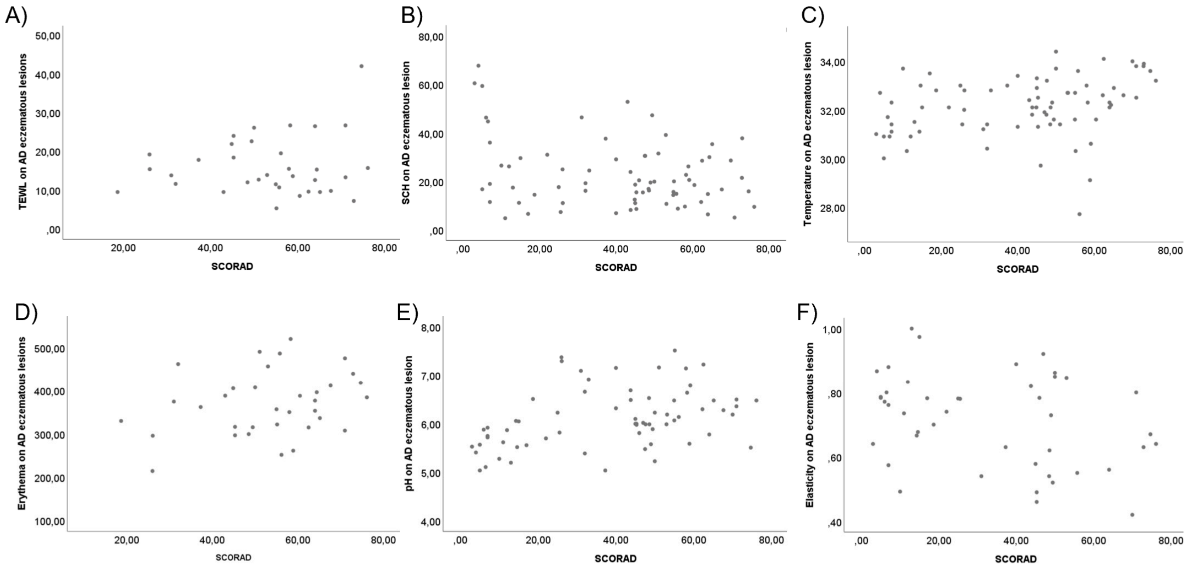

Figure S2. Correlation between disease severity and homeostasis parameters on AD eczematous lesions. (**A**) SCORAD and TEWL (r = −0.016, p = 0.892). (**B**) SCORAD and SCH (r = −0.319, p = 0.006). (**C**) SCORAD and temperature (r = 0.291, p = 0.011), (**D**) SCORAD and erythema (r = 0.258, p = 0.080), (**E**) SCORAD and pH (r = 0.401, $p < 0.001$), (**F**) SCORAD and elasticity (r = −0.419, p = 0.003). AD, atopic dermatitis; SCH, stratum corneum hydration; SCORAD, SCORing Atopic Dermatitis.
